# Synovial IL-9 facilitates neutrophil survival, function and differentiation of Th17 cells in rheumatoid arthritis

**DOI:** 10.1186/s13075-017-1505-8

**Published:** 2018-01-30

**Authors:** Kaustav Chowdhury, Uma Kumar, Soumabha Das, Jaydeep Chaudhuri, Prabin Kumar, Maumita Kanjilal, Parashar Ghosh, Geetabali Sircar, Ravi Kiran Basyal, Uma Kanga, Santu Bandyopadhaya, Dipendra Kumar Mitra

**Affiliations:** 1 Department of Transplant Immunology and Immunogenetics, All India Institute of Medical Sciences (AIIMS), Room No-75, New Delhi, 110029 India; 2 Department of Medicine, All India Institute of Medical Sciences (AIIMS), New Delhi, India; 3 Rheumatology Center, Institute of Post Graduate Medical Education and Research (IPGMER), Kolkata, India; 4 Indian Institute of Chemical Biology, Council of Scientific and Industrial Research (CSIR), Kolkata, India

**Keywords:** Rheumatoid arthritis, Interleukin-9, Neutrophils, Th17 cells

## Abstract

**Background:**

Role of Th9 cells and interleukin-9 (IL-9) in human autoimmune diseases such as psoriasis and ulcerative colitis has been explored only very recently. However, their involvement in human rheumatoid arthritis (RA) is not conclusive. Pathogenesis of RA is complex and involves various T cell subsets and neutrophils. Here, we aimed at understanding the impact of IL-9 on infiltrating immune cells and their eventual role in synovial inflammation in RA.

**Methods:**

In vitro stimulation of T cells was performed by engagement of anti-CD3 and anti-CD28 monoclonal antibodies. Flow cytometry was employed for measuring intracellular cytokine, RORγt in T cells, evaluating apoptosis of neutrophils. ELISA was used for measuring soluble cytokine, Western blot analysis and confocal microscopy were used for STAT3 phosphorylation and nuclear translocation.

**Results:**

We demonstrated synovial enrichment of Th9 cells and their positive correlation with disease activity (DAS28-ESR) in RA. Synovial IL-9 prolonged the survival of neutrophils, increased their matrix metalloprotienase-9 production and facilitated Th17 cell differentiation evidenced by induction of transcription factor RORγt and STAT3 phosphorylation. IL-9 also augmented the function of IFN-γ + and TNF-α + synovial T cells.

**Conclusions:**

We provide evidences for critical role of IL-9 in disease pathogenesis and propose that targeting IL-9 may be an effective strategy to ameliorate synovial inflammation in RA. Inhibiting IL-9 may have wider impact on the production of pathogenic cytokines involved in autoimmune diseases including RA and may offer better control over the disease.

**Electronic supplementary material:**

The online version of this article (10.1186/s13075-017-1505-8) contains supplementary material, which is available to authorized users.

## Background

Helper T (CD4^+^) cells preferentially producing interleukin (IL)-9 have recently been described as distinct Th9 cells and implicated in several inflammatory conditions such as infectious, neoplastic and autoimmune diseases [1–4]. Contribution of these cells in the pathogenesis of autoimmunity has been demonstrated in animal studies of colitis [5, 6], uveitis [7], and experimental autoimmune encephalomyelitis (EAE) [8–10]. However, reports on their presence or enrichment at the pathologic sites and impact on the local inflammatory cascade in human autoimmune diseases are limited, except in psoriasis [11], systemic lupus erythematosus [12], and colitis [6].Very recently, Th9 cells have been identified in rheumatoid arthritis (RA) peripheral blood and synovial tissues and fluid, but their precise role in RA pathology is not depicted conclusively [13, 14]. Therefore, investigating Th9 cells in RA seems to be important in determining the local immune response. Therefore, we hypothesized that Th9/IL-9-producing cells would be abundant in RA synovial fluid (SF). This study is an attempt to determine the status of Th9 cells at the disease site of RA patients and their involvement in disease progression.

Pathogenesis of RA involves a complex inflammatory cascade mediated by massive influx of various effector immune cells, their activation leading to copious production of tissue-damaging proteases like matrix metalloproteinases (MMPs) [15], and collagenase. Cellular infiltrates in RA joints majorly constitute neutrophils and lymphocytes (T and B) [16–19]. Neutrophils and various T cell subsets producing distinct pro-inflammatory cytokines have been demonstrated as key effector cells augmenting the local inflammatory response during active disease. Synovial neutrophils are activated by diverse cytokines derived from infiltrating T cells and macrophages [20–22]. Involvement of Th1 and Th17 cells in RA has been demonstrated earlier [23–27]. Among diverse cytokines present in SF of RA joints, interferon gamma (IFN-γ), tumor necrosis alpha (TNF-α), and IL-17 are believed to be critical mediators of inflammation in RA. IL-17 and TNF-α have been documented to activate the neutrophils to produce MMPs [28, 29] which are responsible for joint destruction. Most recent findings implicate Th17 cell as an important effector in pathogenic process of RA [30]. Elucidating functional hierarchy of distinct effector T cells producing key pathogenic cytokine(s) is critical and may provide novel opportunity for modulating inflammation in RA and disease remission.

Recent evidence suggests the role of Th9 cells in psoriatic skin lesions [7] in enhancing other T cell subsets producing effector cytokines like IFN-γ, IL-17. However, Th9 cells and their role in RA still remain unclear. Therefore, in this study we asked following questions: (i) Do Th9 cells exist in RA-affected synovial milieu in patients as a distinct in vivo T cell subset? (ii) If so, do they exert a hierarchic influence on other inflammatory immune cells accumulated in pathologic sites of RA? (iii) Does synovial IL-9 augment the function of infiltrating T cells and neutrophils thereby potentiating the inflammatory cascade in RA?

Here, we show the enrichment of Th9/IL-9+ cells as a distinct inflammatory T cell subset in the SF of active RA patients. Our study demonstrates that (i) Th9 cells are enriched in SF, (ii) SF IL-9 drives the inflammatory cascade by inhibiting apoptosis of neutrophils and production of MMP-9 and (iii) augments the differentiation of Th17 cells in RA-affected synovia. We reveal a hitherto unknown mechanism by which Th9/IL-9 cells may perpetuate synovial inflammation in RA to the best of our current knowledge.

## Methods

### Study subjects

This study was conducted in treatment-naïve active RA patients, fulfilling the 1987 American College of Rheumatology (ACR) criteria [31]. All RA patients disease activity was measured with 28-joint disease activity score (DAS-28), which is based on the number of tender/swollen joints and the erythrocyte sedimentation rate (ESR). All RA patients had DAS-28 ESR > 5. Out of 28 patients, 20 were female and 8 were male (Additional file 1: Table S1). Fifteen healthy controls (HCs) and 10 osteoarthritis (OA) patients with median age 38.6 ± 8.9 and 43.5 ± 2.98 years respectively were enrolled in this study. The female:male ratios of HC and OA groups were 9:6 and 5:5 respectively. HCs were free from any rheumatologic disorders, acute or chronic ailments such as infections, diabetes mellitus or any other endocrine disorders, liver or renal abnormalities. These were confirmed by clinical as well as appropriate laboratory investigations including radiography, complete blood count (CBC), and serology for HIV and hepatitis, relevant endocrine, renal and hepatic assays. OA patients were diagnosed according to ACR classic clinical criteria (inclusion) supplemented with radiographic evaluation, ESR (<40 mm/hour), white cell count of the synovial aspirate (<1000/μl), rheumatoid factor (RF) titer (<1:40). All of our OA patients presented with involvement of knee joints. This is an ex vivo basic experimental study and it is approved by the institutional ethics committee (All India Institute of Medical Sciences, New Delhi, India, letter no: SH/10/11/TII). All blood and SF samples were collected from RA and OA patients after taking duly signed written consent form.

### Cell preparation and enumeration of T cell frequency

Peripheral blood and SF fluid mononuclear cells (PBMCs and SFMCs) were isolated by Ficol hypaque (Lymphoprep, Axis-Shield, Oslo, Norway) density gradient centrifugation. Cell viability was checked by 0.1% trypan blue dye exclusion test and found to be > 95% viable. Cells were suspended in media RPMI-1640 (Caisson Laboratories, Smithfield, UT USA) supplemented with L-Glutamine, HEPES (Sigma-Aldrich, St. Louis, MO, USA), antibiotics (Biological Industries, Kibbutz Beit-Haemek, Israel) and 10% heat inactivated fetal calf serum (FCS, Biological Industries). PBMCs/SFMCs were stimulated with plate-bound anti-CD3 (Clone: UCHIT, BD Pharmingen, San Jose, CA, USA; 5ug/ml) and anti-CD28 (clone: CD28.6 eBiosciences, San Diego, CA, USA, 2.5ug/ml) for 48 hours in humidified 5% CO_2_ incubator at 37 °C. In some experiments evaluating reagents rIL-9 (20 ng/ml; Abcam, Cambridge, MA, USA), SF (1:9 ratio SF:media), anti-IL-9 monoclonal antibody (10 μg/ml, Abcam) were added in the cell culture to block endogenous IL-9 present in RA SF. Golgi transport inhibitor monensin was added during last 6 hours of culture. For surface staining, cells were washed with phosphate-buffered saline (PBS) and incubated with CD4 phycoerythrin tandem conjugate with cyanine (PE-Cy5) (BD Pharmingen) for 15 minutes on ice. Intracellular cytokine staining for IL-9, IFN-γ, TNF-α, and IL-17A was done after fixation and a permeabilization process using dye-conjugated monoclonal antibodies Anti-IL-9 PE, (BioLegend, San Diego, CA, USA), anti-IFN-γ fluorescein isothiocyanate (FITC), anti-TNF-α PE (BD Pharmingen), anti-IL-17A FITC (eBiosciences). For Th17 cell transcription factor analysis in HC and RA patients, isolated PBMCs and SFMCs were isolated, washed and surface-stained for CD4 (anti-CD4 PECy5, BD Pharmingen) and after fixation and permeabilization, cells were further incubated with anti-retineic-acid-receptor-related orphan nuclear receptor gamma t (RORγt) PE antibody (eBiosciences) for 30 minutes in dark. Data was acquired in FACS caliber (BD, Franklin Lakes, NJ, USA) and analyzed using FlowJo (Tree Star, Ashland, OR, USA).

### Neutrophil isolation and apoptosis

To study the neutrophil apoptosis in the presence of the SF of RA patients, neutrophils were enriched from healthy donor by density gradient centrifugation on polymorphoprep as per the manufacturer’s protocol (Axis-Shield PoC AS) [32]. After lysis of red blood cells (RBCs) neutrophils were washed with PBS (pH 7.4) and suspended in media RPMI-1640 (as described in the previous section). Purity and viability (>95%) of enriched neutrophils were assessed using Leishman staining and the trypan blue dye exclusion method. Neutrophils were identified in FACS by using CD15 as a neutrophil identifying marker [33]. To observe the effect of IL-9 on neutrophil survival, neutrophils (isolated freshly from healthy donor peripheral blood) were cultured in the presence and absence of rIL-9 (20 ng/ml, eBioscience), in media for 12 hours at 37 °C. Neutrophils were harvested, washed twice with PBS, and incubated with anti-CD15 PE and anti-annexin V FITC antibody (BD Pharmingen) for 15 minutes on ice. To determine the effect of endogenous IL-9 on neutrophil survival, neutrophils from RA SF (1 × 10^6^) were cultured in the presence of rIL-9, autologous SF (100 μl SF of active RA patient in 400 μl RPMI-1640) and anti-IL-9 antibody (10 μg/ml; Abcam). Cells were harvested after 12 hours and washed twice with PBS. Cells were subjected to FACS-based staining for CD15 and annexin V and acquired by FACSCalibur (BD). Data analysis was done with FlowJo X (Tree Star).

### Neutrophils activation and IL-9 receptor expression

To study the neutrophil activation and function, freshly isolated healthy neutrophils (1 × 10^6^) were stimulated with rIL-9 (eBioscience), lipopolysaccharide (LPS) (5 ng/ml, Sigma-Aldrich) and LPS plus rIL-9 for 12 hours. Cells were then incubated with anti-CD69 PE antibody (BD Pharmingen) and anti-IL-9 receptor (anti-CD129) PE antibody (BioLegend) on ice for 15 minutes on ice.

### MMP-9 production by neutrophils

To determine the matrix metalloproteinase-9 (MMP-9) production by neutrophils, isolated neutrophils (1 × 10^6^) from healthy controls were stimulated either with RA SF (cell-free SF, DAS-28 score > 5, 1:9 ratio SF:media), rIL-9 (20 ng/ml) or anti-IL-9 blocking antibody (10 μg/ml, Abcam) for 12 hours and monesin was added (as a golgi transport inhibitor, Sigma-Aldrich) during last 6 hours of culture. Neutrophils were fixed with 2% formaldehyde, permeabilized and incubated with rabbit anti-human anti-MMP-9 primary antibody (1:250 dilution, Santa Cruz Biotechnology, Dallas, TX, USA) for 30 minutes at room temperature followed by secondary anti-rabbit IgG FITC (dilution 1:100; Abcam) for another 30 minutes at room temperature. Stained samples were acquired by FACSCalibur (BD) and analyzed with Flow Jo (Tree Star).

### Western blot for MCL-1 and STAT3 phosphorylation

Neutrophils were enriched from SF of RA patients and cultured (2 × 10^6^) as mentioned above in apoptosis assay. After 12 hours of culture, cells were harvested and were lysed in lysis buffer [protenase inhibitor cocktail (BD Pharmingen) and phosphoSTOP (Sigma-Aldrich) present in lysis buffer along with radioimmunoprecipitation assay buffer (RIPA, Cell Signaling Technology, Danvers, MA, USA)]. After extracting proteins from each culture condition (media, SF, rIL-9, anti-IL-9), concentration was measured with protein estimation kit (Bio-Rad, Hercules, CA, USA). Each protein sample (50 μg) was subjected to SDS-polyacrylamide gel electrophoresis (PAGE). Proteins were transferred to polyvinylidene difluoride membranes (PVDF, EMD Millipore, Billerica, MA, USA) and western blot was performed using anti-induced myeloid leukemia cell differentiation protein [anti-induced myeloid leukemia cell differentiation protein (MCL-1) monoclonal antibody, 1:500 dilution, Santa Cruz Biotechnology, Dallas, TX, USA]. Beta-actin (anti-β-actin, 1:500 dilutions, Santa Cruz Biotechnology) was used as loading control. Detection of specific protein was done by Luminata Forte HRP substrate (EMD Millipore). To determine signal transducer and activator of transcription 3 (STAT3) phosphorylation in CD4+ T cells, freshly isolated PBMCs (2 × 10^6^) from healthy individuals were enriched for CD4+ T cells with the help of magnetic enrichment kit (Miltenyi Biotec, San Diego, CA, USA). Enriched CD4+ cells (98% purity, viability was 97.8% with trypan blue dye exclusion method) were cultured in presence of rIL-9 (20 ng/ml, eBiosciences), SF of RA patient (DAS28-ESR > 5+, 100 μl SF in 500 μl RPMI1640) and anti-IL-9 polyclonal goat antibody (20 Abcam) for 12 hours. Cells were harvested and cell lysate was prepared in lysis buffer (mentioned above). After extracting proteins from each culture condition (media, anti-CD3/28, rIL-9, RA SF) protein concentration was measured with protein estimation kit (Bio-Rad). Each protein sample (50 μg) was subjected to SDS-PAGE. Proteins were transferred to polyvinylidenedifluoride membranes (PVDF, EMD Millipore) and western blot was performed anti-phospho STAT3 (Tyr705, 1:1000 dilution, Cell Signaling Technology) antibody. Detection of specific protein was done by Luminata Forte HRP substrate (EMD Millipore).

### Detection of STAT3 co-localization by confocal microscopy

To determine the STAT3 phosphorylation and cellular localization, freshly isolated PBMCs (2 × 10^6^) from healthy subjects were stimulated as per same culture conditions mentioned above for STAT3 western blot. Cells were harvested after 12 hours and washed twice with PBS. Surface staining was done with anti CD4 FITC (BD Pharmingen) incubation on ice for 15 minutes. After fixing and permealization, cells were incubated in dark with DAPI-conjugated Prolong Gold anti-fade reagent as per the manufacturer’s protocol (Invitrogen, Waltham, MA, USA) and anti-phopho-STAT3 APC antibody (Tyr705, 1:300 dilution, Lifespan Biosciences, Seattle, WA, USA) at room temperature for 30 minutes. Cells were then observed under a Plan Apo VC 60X/1.40 oil objective on an inverted Eclipse Ti Nikon microscope equipped with a QImaging-Rolera EMC^2^ camera to capture image (Nikon, Tokyo, Japan).

### Cytokine ELISA

Levels of soluble IL-9 and MMP-9 were assessed in plasma (HC, OA, RA) and SF (RA and OA) by ELISA kit IL-9 (Abcam) and MMP-9 (eBioscience) respectively, as per the manufacturer’s protocol.

### Statistical analysis

Statistical analysis was done with GraphPad prism5 software (La Jolla, CA, USA) by using Student’s *t* test for unpaired samples. One-way ANOVA, Dunnett’s multiple comparison tests was used for statistical significance evaluation in cytokine production study in different conditions. The data were presented as mean ± SEM and values *p* < 0.05 was considered significant.

## Results

### Enrichment of Th9 cells in the RA SF correlates with disease activity score (DAS28-ESR)

**Fig. 1:**
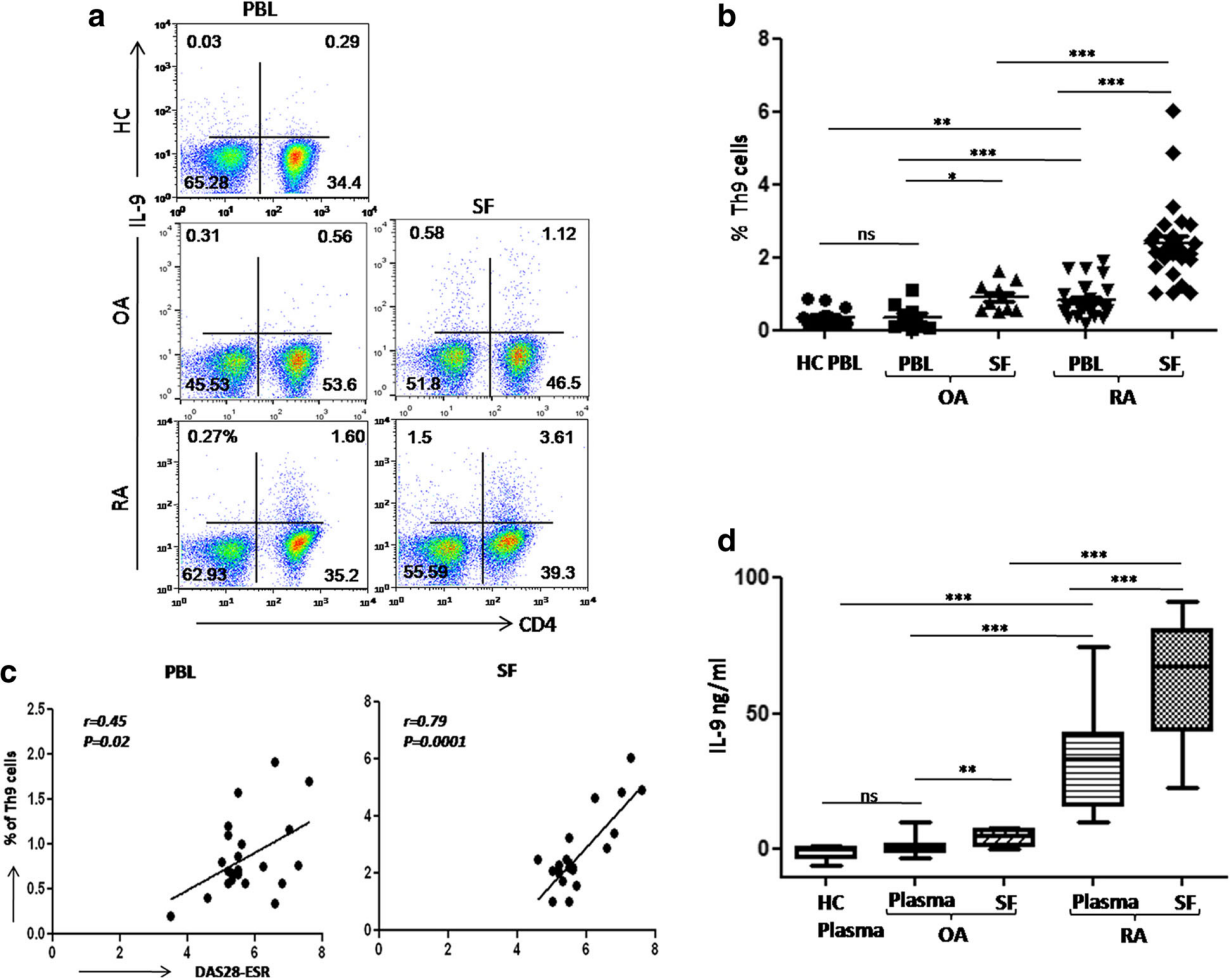
Th9 cell frequency is higher in SF of RA patients. **a** Representative FACS plots show Th9 cell frequency in PBL (HC, OA, and RA) and SF (OA and RA). **b** Cumulative scatter plot shows frequency of Th9 cells in of RA (PBL and SF; n = 28), OA (PBL and SF; n = 10), HC (PBL; n = 15), significant enrichment of Th9 cells in SF of RA patients. **c** Significantly higher DAS28-ESR correlation with Th9 cell frequency in RA SF (r = 0.79, *p* = 0.0001) and RA PBL (r = 0.45, *p* = 0.02, n = 28). **d** IL-9 ELISA performed in plasma (HC; n = 15, OA; n = 10, RA; n = 20) and RA SF (n = 20), shows higher levels of soluble IL-9 in RA SF 9pt?>compared to autologous RA plasma (mean ± SEM, *p < 0.05, **p < 0.005, ***p < 0.0001). *DAS28* disease activity score of 28 joints, *ESR* erythrocyte sedimentation rate, *IL* interleukin, *OA* osteoarthritis, *PBL* peripheral blood, *RA* rheumatoid arthritis, *SF* synovial fluid, *Th* T helper

We aimed to enumerate the frequency of IL-9-producing CD4+ T cells in peripheral blood (PBL) and SF of RA patients by flow cytometry. Frequency of Th9 cells was elevated in the PBL of RA patients compared to HC and OA patients. However, it was not significantly higher in the PBL of OA patients compared to HC. Interestingly, frequency of Th9 cells was markedly higher in RA SF relative to their autologous PBL as well as to their disease control group OA SF, suggesting their enrichment at the pathologic site (RA SF, Fig. 1a and b). Th9 cell frequency of both PBL (r *=* 0.45; *p =* 0.02) and SF (r *=* 0.79; *p =* 0.0001) correlated with DAS-28-ESR disease activity score of the patients (Fig. 1c). Soluble level of IL-9 was also significantly elevated in RA PBL and SF compared to OA PBL and SF (Fig. 1d).

### Synovial IL-9 of RA patients inhibits apoptosis of neutrophils

**Fig. 2:**
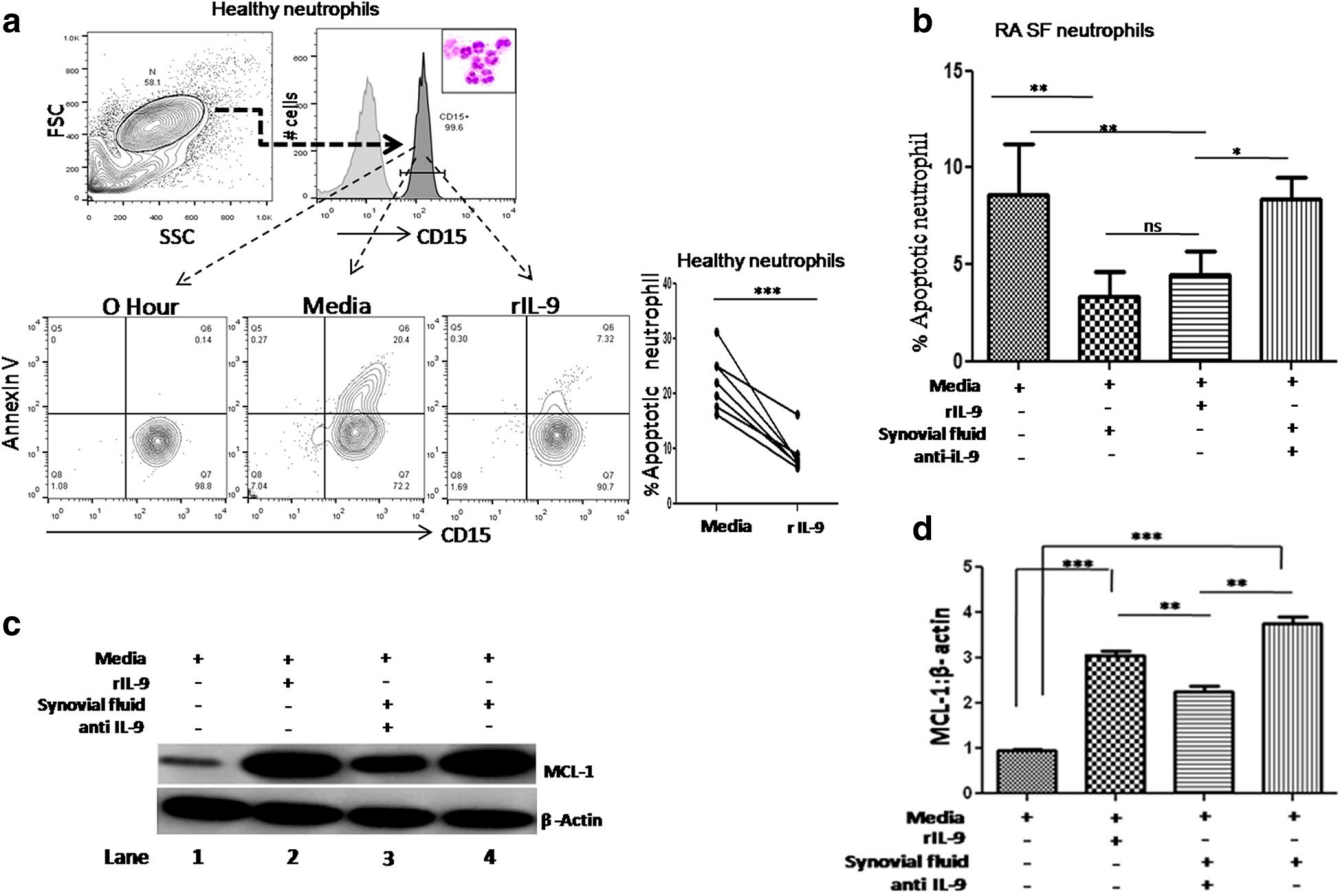
IL-9 provides survival to RA SF neutrophils. **a** FACS plots show reduced annexin V on CD15-gated neutrophils (*upper panel*, Leishman’s stain image of neutrophils in inset) in presence of rIL-9 and cumulative data is shown in line graph (*lower right panel*, n = 7). **b** Cumulative bar graph shows reduction in apoptosis of RA SF-derived neutrophils in the presence of RA SF and rIL-9, addition of anti-IL9 antibody in RA SF increased apoptosis (n = 8, mean ± SEM; *p* < 0.006). **c** Western blot image shows MCL-1 expression in SF-derived neutrophils under different conditions (media, rIL-9, anti-IL-9, RA SF). MCL-1 increases with rIL-9 (*lane 2*), RA SF (*lane 4*) and reduces upon blocking of endogenous IL-9 present in RA SF (*lane no 3*). Data are representative of two different experiments performed. **d** Relative (MCL-1/β-actin) densitometry bar graph of the experiment (n = 3, mean ± SEM, unpaired *t* test, *p < 0.05,**p < 0.001,***p < 0.0001). *IL* interleukin, *IL-9R* interleukin 9 receptor, *MCL-1* induced myeloid leukemia cell differentiation protein, *RA* rheumatoid arthritis, *SF* synovial fluid, *Th* T helper

We investigated the impact of endogenous IL-9 of RA SF on the survival and activation of synovial neutrophils, the most abundant cells infiltrating in the RA joints [34]. Addition of recombinant IL-9 (rIL-9) significantly reduced the apoptosis of healthy neutrophils in vitro as measured by the annexin V staining (Fig. 2a). To understand it is in vivo relevance, we measured the spontaneous apoptosis of RA SF derived neutrophils in presence and absence of SF. RA SF, and rIL-9 significantly reduced, while blocking IL-9, increased the spontaneous apoptosis of neutrophil (Fig. 2b and Additional file 2: Figure S1). rIL-9 increased the expression of anti-apoptotic protein, MCL-1 (a BCL-2 homolog) in RA SF-derived neutrophils. Addition of RA SF increased the expression of MCL-1, even higher than rIL-9 alone. Moreover, blocking endogenous IL-9 in SF reduced the expression of MCL-1 (Fig. 2c, d). Therefore, we concluded that IL-9 present in the SF of RA patients inhibits the apoptosis and might allow them to cause prolonged tissue damage.

### IL-9 activates neutrophils and enhances their matrix metalloproteinase production

**Fig. 3:**
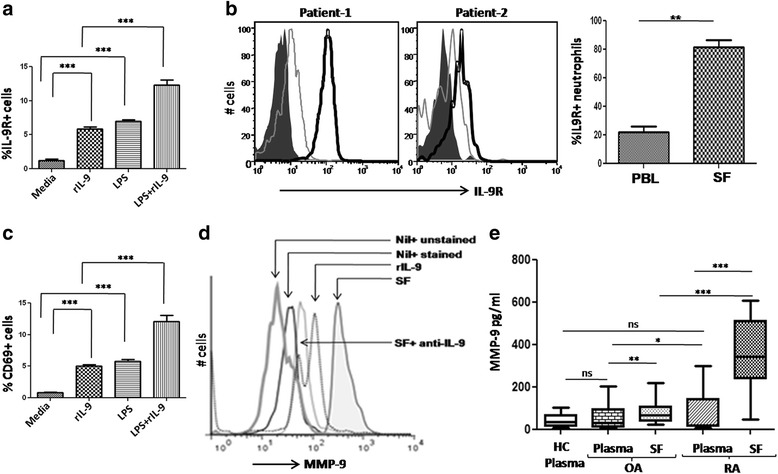
Effect of IL-9 on neutrophil activation and IL-9R expression. **a** Cumulative bar graph shows IL-9R on neutrophils under different stimulation (rIL-9, LPS, LPS + rIL-9, n = 6, mean ± SEM). **b** Histogram plot shows higher expression of IL-9R on SF-derived (*dark open line*) neutrophils than PBL neutrophils of RA patients (*light open line*, *dark area* is control isotype), cumulative bar graph shows IL-9 receptor expression on neutrophils of RA patients; PBL and SF, n = 7).**c** CD69 expression on neutrophils under different culture conditions (rIL-9, LPS, LPS + rIL-9, n = 6, mean ± SEM). **d** One representative FACS histogram plot of six individual experiments shows intracellular MMP-9 in the presence of rIL-9 (*light open line*), SF (*dark open line*, untreated in *dark shaded area*). **e** Soluble MMP-9 level in plasma (HC; n = 15, OA; n = 10, RA; n = 17) and SF (OA; n = 10, RA; n = 17, unpaired *t* test, mean ± SEM *p < 0.05, **p < 0.001, ***p < 0.0001). *HC* healthy control, *IL-9R* interleukin 9 receptor, *LPS* lipopolysaccharide, *MMP-9* matrix metalloproteinase-9, *OA* osteoarthritis, *RA* rheumatoid arthritis, *SF* synovial fluid

Enhanced survival of neutrophils prompted us to investigate the impact of IL-9 on their activation status. rIL-9 could induce IL-9 receptor [CD129/interleukin 9 receptor (IL-9R)] on neutrophils. However, LPS activated healthy neutrophils expressed higher levels of IL-9 receptor, suggesting activation dependence of its expression (Fig. 3a). Similarly, IL-9 receptor was higher on RA SF-derived neutrophils compared to their autologous PBL-derived neutrophils (Fig. 3b). rIL-9 also induced surface expression of CD69, this suggests IL-9 can activate neutrophils (Fig. 3c). MMP-9 is a protease involved in the pathogenesis of RA [35]. Endogenous IL-9 present in the SF of RA patients and rIL-9 both enhanced MMP-9 production by neutrophils derived from healthy individuals. Whereas blocking endogenous IL-9 with anti-IL-9 antibody in RA SF decreased the production of MMP-9 in neutrophils (Fig. 3d). Moreover, the soluble level of MMP-9 was also significantly higher in RA (SF and plasma) than in OA (SF and plasma, Fig. 3e).

### IL-9 potentiates functional differentiation of Th17 cells

**Fig. 4:**
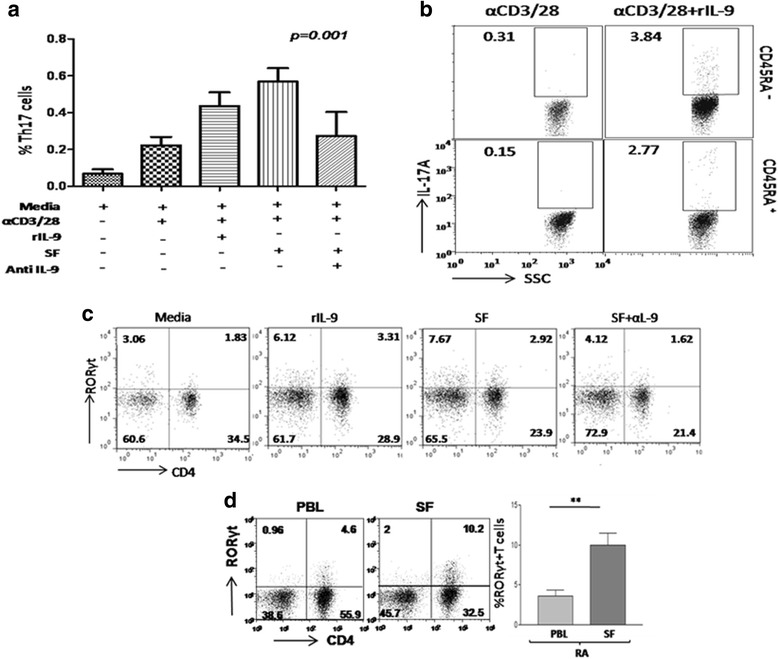
IL-9 promotes differentiation of Th17 cells. **a** Bar graph shows rIL-9, RA SF can increase level of IL-17A in CD4+ (Th17) cells from healthy donor, decreased upon blocking with anti-IL-9 in RA SF (n = 6, one-way ANOVA analysis with Dunnett’s multiple comparison test is applied for statistical significance, *p < 0.05). **b** Representative FACS dot plots show frequency of IL-17A^+^ cells in both naïve (CD4 + CD45RA^+^) and memory (CD4 + CD45RA^-^) T cell compartment (n = 3). **c** FACS plots show frequency of RORγt + T cells in healthy PBMCs in presence of rIL-9, RA SF, anti-(CD3 + 28). Addition of IL-9 blocking antibody in RA SF reduced the expression of RORγt (n = 5). **d** FACS plots show RORγt^+^CD4^+^ T cells in RA PBL and RA SF, cumulative bar graph shows RORγt^+^CD4^+^ T cells in RA PBL and SF (n = 10, unpaired *t* test, mean ± SEM, **p < 0.005). *IL* interleukin, *IL-9R* interleukin 9 receptor, *PBL* peripheral blood, *RA* rheumatoid arthritis, *RORγt* retineic-acid-receptor-related orphan nuclear receptor gamma t, *SF* synovial fluid, *Th* T helper

Increased frequency of synovial Th9 cells and its correlation with the disease activity score (DAS28-ESR) prompted us to investigate the impact of IL-9 on differentiation of Th17 cells. rIL-9 increased the number of IL-17A+ CD4+ T cells in healthy PBMCs stimulated in vitro with TCR engagement especially in memory (CD45RA-) T cells (Fig. 4a, b). This hints toward an IL-9-dependent Th17 differentiation of memory T cells in the synovium of RA patients. We further looked for Th17 differentiation-related transcription factor, Retinoic acid-related orphan receptor γt (RORγt) in presence of endogenous and synthetic IL-9. To this end, we have observed substantial increase in the number of RORγt^+^ T cells in the presence of both rIL-9 and RA SF (Fig. 4c). This is further substantiated by the presence of a higher number of RORγt^+^ T cells (gated on CD4+ cells) in RA SF compared to RA PBL (Fig. 4d). All together, these findings suggest that endogenously produced IL-9 present in RA SF can potentiate differentiation of Th17 cells.

### Synovial IL-9 potentiates T cells present in the RA-affected joints

**Fig. 5:**
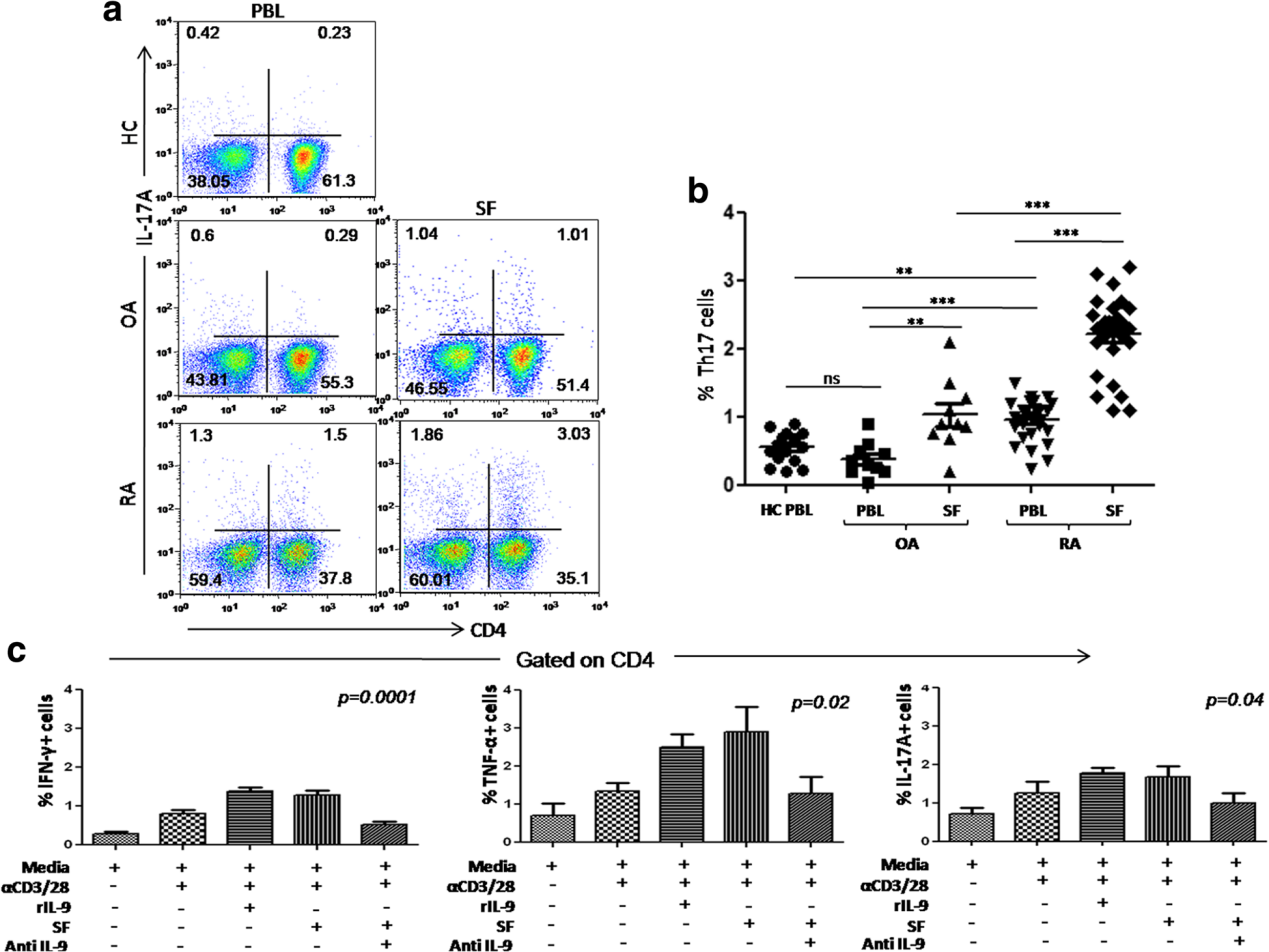
IL-9 induced differentiation of Th17 and other effector T cells. **a** Representative FACS plots show Th17 cell frequency in PBL (HC, OA, RA) and SF (OA and RA).**b** Cumulative scatter plot shows frequency of Th17 (CD4 + IL-17A+) in RA (PBL and SF, n = 26), OA (PBL and SF, n = 7), HC PBL (n = 12). **c** Cumulative bar graphs show IFN-γ + (*p* = 0.0001) and TNF-α + (*p* = 0.02), IL-17A+ (*p* = 0.04) CD4+ cells were significantly higher in the presence of rIL-9 or RA SF, blocking IL-9 abrogated the same (n = 6, one-way ANOVA analysis with Dunnett’s multiple comparison test is applied for statistical significance, *p < 0.05). *HC* healthy control, *IL-9R* interleukin 9 receptor, *IFN-γ* interferon gamma, *OA* osteoarthritis, *RA* rheumatoid arthritis, *SF* synovial fluid, *Th* T helper

To observe if IL-9 can differentiate IFN-γ^+^, TNF-α^+^, and IL-17A^+^ effector T cells, we studied the effect of rIL-9 and RA SF on their frequency in SF of RA patients. The frequency of Th17 cells was significantly higher in the peripheral blood of RA patients compared to that of HC and OA patients (Fig. 5a, b). Th17 cells were even more abundant in the RA SF compared to OA SF, indicating their possible enrichment in the pathogenic milieu (Fig. 5b).To validate this further we stimulated T cells from RA SF in presence of rIL-9 and RA SF. T cells obtained from RA SF showed significantly higher frequency of IFN-γ^+^, TNF-α^+^, and IL-17A^+^ cells in presence of rIL-9 and RA SF. Moreover, neutralizing IL-9 of SF by blocking antibody significantly reduced their frequencies (Fig. 5c). Interestingly, both the basal and IL-9-induced frequencies of these cytokine-producing T cells were much higher in the SF of RA patients relative to their peripheral compartment (data not shown). This suggests IL-9 mediated potentiation of inflammatory cytokine-producing T cells. This is further supported by higher expression of IL-9 receptors on these effector T cells, both in peripheral and SF (data not shown).

### IL-9 of SF of RA patients induces STAT3 phosphorylation and nuclear translocation

**Fig. 6:**
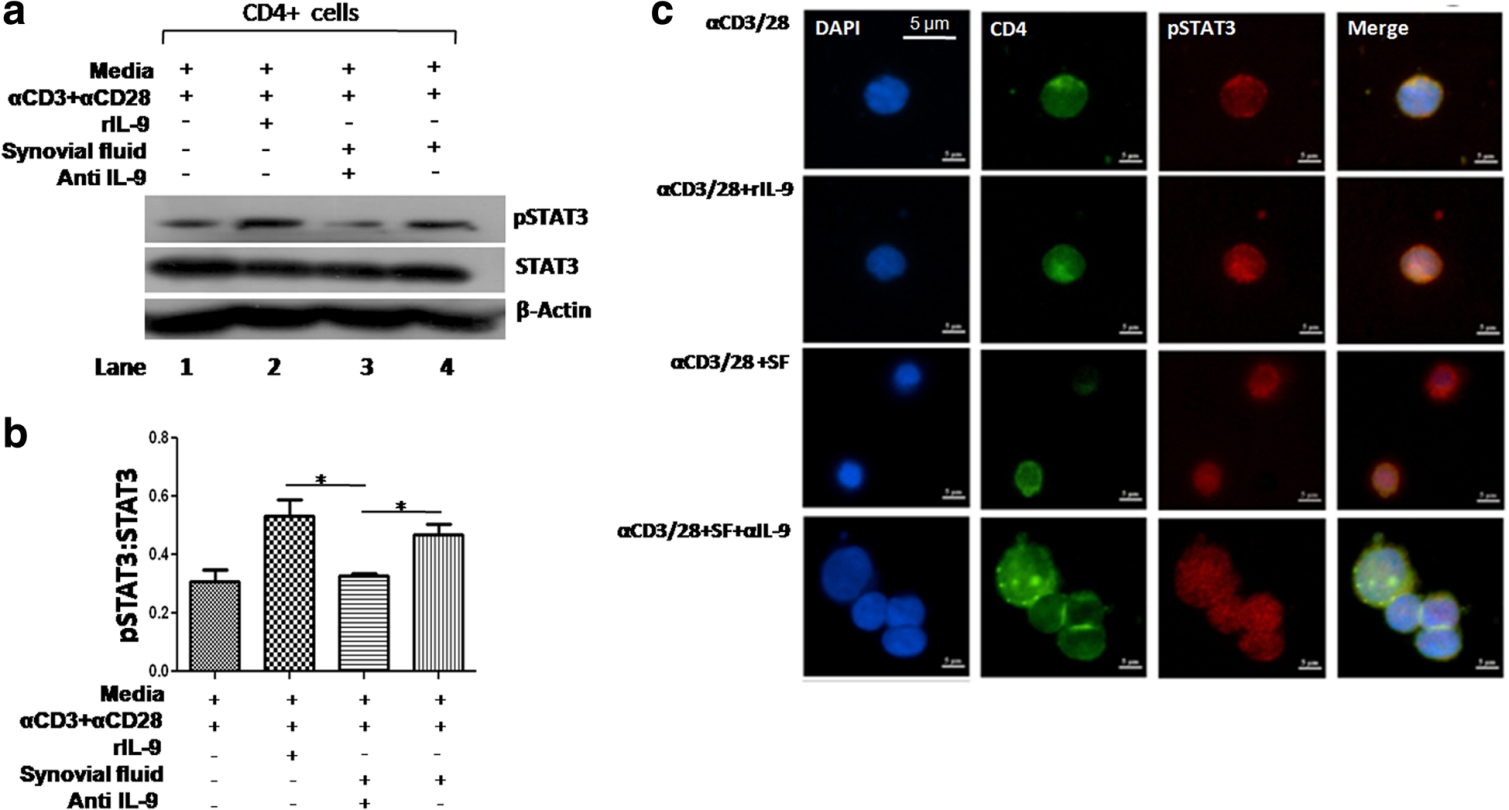
IL-9 induces transcription factor for Th17 cells. **a** Representative western blot image shows phosphorylation of STAT3 (pSTAT3) in the presence of rIL-9 and SF. pSTAT3 was enhanced with rIL-9 and SF (*lane 2 and 4*) and in presence of anti-IL-9 antibody in SF reduced pSTAT3 (*lane 3*, n = 2). **b** Bar graph shows cumulative densitometry of pSTAT3 (pSTAT3:STAT3, β-actin as loading control, n = 5, unpaired *t* test, mean ± SEM, *p < 0.05).**c** Representative confocal micrograph from two independent experiments, shows phosphorylation of pSTAT3 (*red*), nucleus (DAPI, *blue*) in CD4+ T cells (*green*) and nuclear translocation (merged image in *violet*). *IL-9R* interleukin 9 receptor, *SF* synovial fluid, *STAT3* signal transducer and activator of transcription 3, *α IL-9* anti-interleukin 9

To delineate the signaling pathway responsible for Th17 cell differentiation, we studied STAT3 phosphorylation of healthy donor-derived CD4+ T cells enriched by magnetic sorting in the presence of both recombinant and endogenous IL-9 produced in SF of RA patients. rIL-9 and SF of RA patients could phosphorylate the STAT3 and anti-IL-9 addition in RA SF abrogated the same, suggesting that IL-9 of SF indeed triggers STAT3 phosphorylation, a key transcription factor toward Th17 differentiation (Fig. 6a, b). This is further confirmed by confocal microscopy, showing STAT3 phosphorylation and its nuclear translocation in the presence of IL-9 (Fig. 6c). Taken together, our results suggest IL-9 mediated triggering of the signaling event in leading to Th17 cells differentiation in RA.

## Discussion

Effector T lymphocytes producing various cytokines like IFN-γ, TNF-α, and IL-17 are involved in the pathogenesis of RA [36]. However, the role of Th9 cells in RA has not been studied conclusively so far. Th9 cells were identified as discrete functional T cell subset and initially observed in various Th2-associated immunopathologic conditions such as parasitic infestation and more elaborately studied in allergic asthma, both in mice as well as human [37, 38]. Their differentiation from naïve T cells under the influence of TGF-β and IL-4 was marked by signature transcription factor PU.1 and copious production of IL-9 [39]. Until the recent past, it was thought that these cells belong to the Th2-associated lineage. Several recent animal studies indicate the role of these unique T cell subsets in promoting various autoimmune conditions mediated by pro-inflammatory cytokines like IFN-γ, IL-17. In EAE, Th9 cells have been observed in the lesion sites and IL-9 aggravated the disease severity in EAE [10]. Likewise, in colitis and myasthenia gravis IL-9 has been demonstrated to potentiate the inflammatory pathologies [5, 6, 40]. These results based on animal models indicate a critical role of Th9 cells in the pathogenesis of autoimmune diseases. However, similar reports on IL-9, particularly Th9 cells in human autoimmune conditions are limited, except a few notable very recent studies in psoriasis, colitis, and RA [5, 6, 11, 13]. Schlapbach et al. demonstrated the presence of discrete Th9 cells in the skin, of psoriasis patients, an autoimmune skin proliferative pathology involving various T effector cells. The cells could augment the inflammatory cytokine production by other T cells. Recently, Gerlach et al. identified PU.1 expressing Th9 cell in the lamina propria of ulcerative colitis patients. Ciccia et al. reported that Th9 cells are enriched in synovium of RA patients. These reports not only substantiate the presence of Th9 cells in the pathologic sites, but also suggest their crucial role in autoimmune disease in human.

RA is an autoimmune pathology affecting joints and involves Th1-associated cytokines such as IFN-γ, TNF-α, and Th17-secreted IL-17, etc. In fact, anti-TNF-α therapy in RA patients validates that indeed inflammatory cytokines are involved in the pathogenesis of RA [41]. Here, we show enrichment of Th9 cells in the SF of RA patients, which correlates with disease severity based on DAS-28 ESR scores. Moreover, we demonstrate that rIL-9 as such, but importantly, endogenously produced IL-9 in SF potentiates the differentiation of Th17 cells, as evidenced by (i) expression of signature transcription factor RORγt and increase in IL-17 producers in the SF of RA, (ii) blocking IL-9 present in SF of RA patients abrogates such effects in our experiments. We report similar impact of synovial IL-9 of RA patients on the effector T cells producing TNF-α, which is critically involved in the pathogenesis of RA [41].

In the present study, we evaluated the impact of locally enriched IL-9 in RA, and showed that SF IL-9 enhances the survival and activation of neutrophils, evidenced by reduced annexin V expression with concomitant increase of survival protein, MCL-1, and MMP-9 production. Interestingly, MCL-1 upregulation is more pronounced with autologous SF than rIL-9 indicates that other factors including IL-9 may be responsible for delayed apoptosis of neutrophils from RA SF. We demonstrate the potentiating influence of synovial IL-9 on the IL-17+ CD4 T cells derived from RA affected joints, evidenced by their increased production of IL-17(Fig. 5c), upregulation of Th17 related transcription factor expression RORγt (Fig. 4c) and phosphorylation of STAT3 with nuclear translocation (Fig. 6a, c). This is accordance with Elyaman et al., showing IL-9 induces differentiation of Th17 cells in mice [42]. In similar line recently Kundu-Raychaudhuri et al. described IL-9 as a local growth factor for T cells present in RA SF [14]. Therefore, we conclude that enrichment of Th9 cells (and IL-9) in SF facilitates two critical cellular elements involved in augmenting the joint inflammation. Here, we reveal a hitherto unknown interaction between Th9 cells and neutrophils, which augments joint inflammation in RA. This mechanism may contribute to autoimmune diseases in general, thus necessitating further studies in other autoimmune diseases, particularly where Th9 cells are already reported. Previously, Martin et al. reported that activated neutrophils could recruit Th17 cells, which facilitate neutrophilic infiltration driven by chemokine-receptor interaction in autoimmune diseases such as Crohn’s disease and RA [33]. We demonstrate that enriched Th9 cells or IL-9 in SF of RA patients can augment neutrophils activation, survival, and Th17 cell differentiation. This is supported by recent findings of Patricia et al. showing that IL-17 activated neutrophils to kill the *Aspergillus fumigatus* in fungal keratitis [43]. We propose, that enriched Th9 cells possibly trigger and aggravate the synovial inflammation by augmenting Th17 cells and neutrophil survival and activation, thus setting the vicious circuit of their mutual enrichment and activation in the pathologic site of RA. Th9 cells (or IL-9) may be a proximal event in the inflammatory cascade of RA and probable hierarchy of IL-9 may be due to its enhancing impact of synovial T cell function and IL-17 production. Among diverse cytokines present in the SF of RA, possible hierarchy of any of them, particularly newly identified IL-9 may be important in terms of controlling the cascade of inflammation. We have also observed upregulation of rIL-9 receptor expression on IFN-γ, TNF-α, IL-17-producing CD4+ T cells from SF of RA patients (data not shown). Even rIL-9 promoted proliferation of these cytokine-producing cells from RA SF (data not shown). Moreover, enrichment of Th9 cells in the SF may be favored by various IL-1 family cytokines, abundantly present in the affected joints of RA patients [44, 45]. Novel aspects of our study are (i) functionally distinct Th9 cells are enriched in the affected joints of RA patients, (ii) endogenously produced synovial IL-9 prolongs neutrophil survival, their activation, and production of MMPs to perpetuate the inflammation of the affected joints of RA, (iii) IL-9 also potentiates inflammatory cytokine producing T helper cells like Th1, Th17, and TNF-α producer (iv) synovial enrichment of Th9 cells correlates with disease severity. The importance of our finding not only lies in elucidating the Th9-mediated mechanism of inflammation in RA, but also in possible therapeutic translation, given its centrality in augmenting different arms of inflammation involving different cell lineages. Last but not the least, is the positive correlation between Th9 cells and the disease activity in RA (DAS28-ESR). This may be utilized as a disease-monitoring signature for clinical management of autoimmune diseases.

## Conclusions

Overall, our findings demonstrate the relevance of Th9 cells and IL-9 in the pathogenesis of RA in human. Our results bear several translational implications in the clinical management of RA patients. First, the frequency of Th9 cells, particularly in the SF, may serve as a signature biomarker of disease severity and help monitoring patients and therapeutic response. Second, neutralizing IL-9, especially in the affected joints may inhibit the inflammatory process by suppressing the infiltrating neutrophils (survival and activation) and effector T cells (cytokine production). Third, plausible hierarchy of Th9 cells may even have wider impact on the production of pathogenic cytokines involved in autoimmune diseases including RA and may offer better control over the disease. Therefore, targeting IL-9 may be useful for treating RA patients.

## Additional files

Additional file 1: Table S1. Demographic and clinical characteristics of RA patients (n = 28). (DOCX 12 kb)

Additional file 2: Figure S1. Identification and survival of synovial fluid neutrophils in presence of IL-9. Representative gating strategy for FACS plots are showing neutrophils from RA SF. Isolated neutrophils from RA SF were identified with positive staining for CD15 (*dark area* of FACS histogram, *light-shaded area* is isotype control, *right upper panel* FACS plot shows apoptosis of RA SF neutrophils at 0 hour). Apoptosis of neutrophils was measured with Annexin V staining in different culture conditions (media, SF, rIL-9 and anti-IL-9, FACS *dot plots* of *lower panel*). (TIF 360 kb)

## Abbreviations

ACR: American College of Rheumatology
ANOVA: Analysis of variance
DAS28: Disease activity score of 28 joints
ELISA: Enzyme-linked immunosorbent assay
ESR: Erythrocyte sedimentation rate
FITC: Fluorescein isothiocyanate
HC: Healthy control
IFN-γ: Interferon gamma
IL: Interleukin
IL-17: Interleukin 17, IL-9R, Interleukin 9 receptor
LPS: Lipopolysaccharide
MCL-1: Induced myeloid leukemia cell differentiation protein
MMP-9: Matrix metalloproteinase-9
OA: Osteoarthritis
PBL: Peripheral blood
PBMC: Peripheral blood mononuclear cell
PBS: Phosphate-buffered saline
PE: Phycoerythrin
PE-Cy5: Phycoerythrin tandem conjugate with cyanine
RA: Rheumatoid arthritis
RIPA: Radioimmunoprecipitation assay
RORγt: Retineic-acid-receptor-related orphan nuclear receptor gamma t
SF: Synovial fluid
SFMC: Synovial fluid mononuclear cell
STAT3: Signal transducer and activator of transcription 3
Th: T helper
TNF-α: Tumor necrosis factor alpha
α IL-9: Anti-interleukin 9
