# Supplementary material for: Synovial IL-9 facilitates neutrophil survival, function and differentiation of Th17 cells in rheumatoid arthritis
Source: Arthritis Res Ther. 2018 Jan 30;20:18. doi: 10.1186/s13075-017-1505-8 (PMC5791733; doi:10.1186/s13075-017-1505-8)
Supplement: Supplementary file 1 — Demographic and clinical characteristics of RA patients (n = 28). (DOCX 12 kb) [file 13075_2017_1505_MOESM1_ESM.docx]

Additional file 1

Table 1

| Characteristic | Values |
| --- | --- |
| Number of patients | 28 |
| Age, mean ± SD (in years) | 40±10.6 |
| Sex ratio, No. male/No. Female | 8/20 |
| Disease duration, mean ± SD (in years) | 16±12 |
| ESR ,mean ±SD mm/hour | 40±7.6 |
| No. RF positive/No. RF negative | 28/0 |
| No. of Swollen joints, mean ± SD | 11±4.2 |
| No. of Tender joints, mean ± SD | 12±4.1 |
| DAS28-ESR, mean ± SD | 5.6±0.85 |
| Methotrexate | 0 |
| Steroids | 0 |
| Biologics | 0 |
| ESR=Erythrocyte sedimentation rate,DAS28= Disease activity score in 28 joints | |

Demographic and clinical characteristics of RA patients
