# Supplementary figures and images for: Synovial IL-9 facilitates neutrophil survival, function and differentiation of Th17 cells in rheumatoid arthritis
Source: Arthritis Res Ther. 2018 Jan 30;20:18. doi: 10.1186/s13075-017-1505-8 (PMC5791733; doi:10.1186/s13075-017-1505-8)

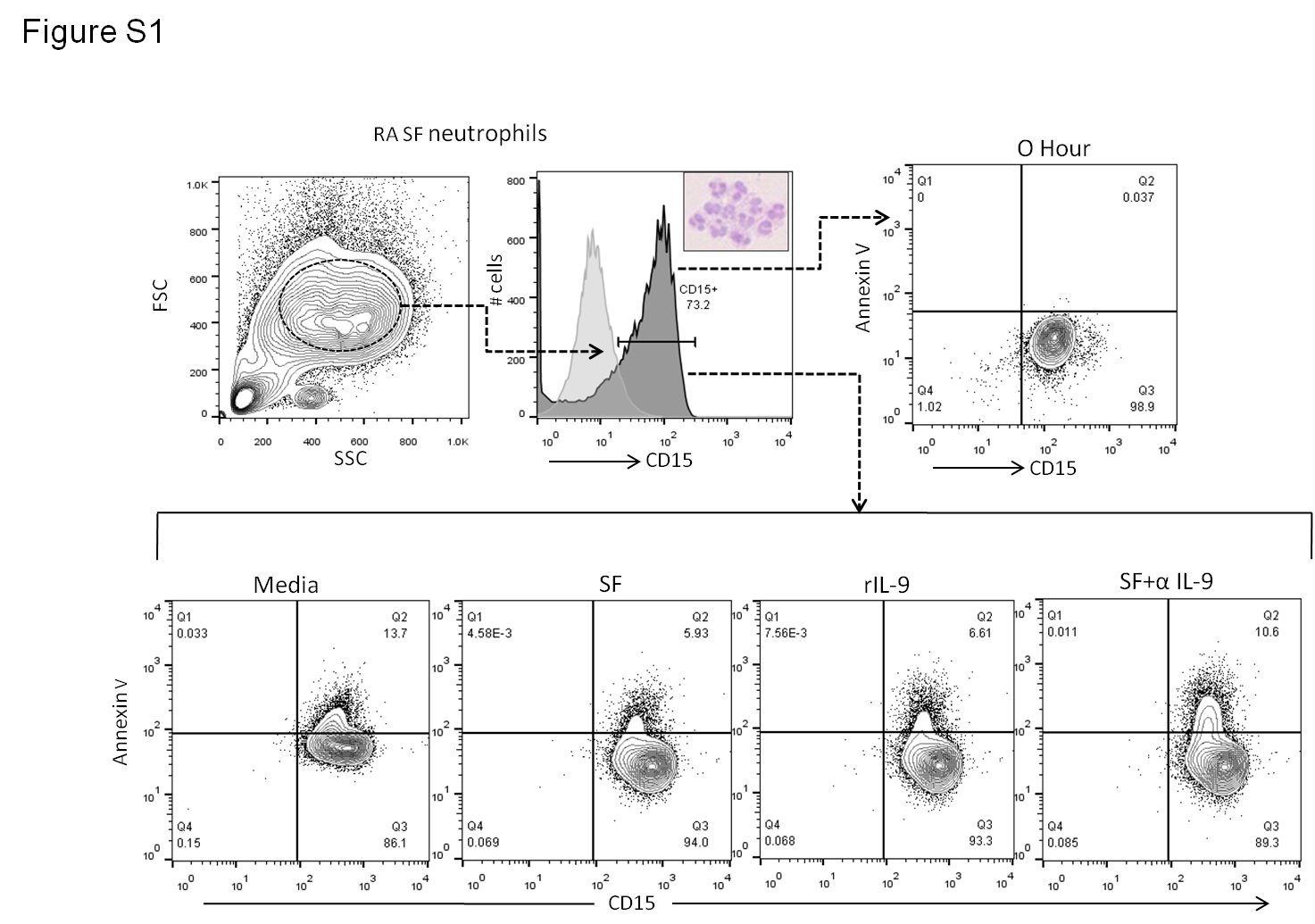

Supplement: Supplementary file 2 — Identification and survival of synovial fluid neutrophils in presence of IL-9. Representative gating strategy for FACS plots are showing neutrophils from RA SF. Isolated neutrophils from RA SF were identified with positive staining for CD15 (dark area of FACS histogram, light-shaded area is isotype control, right upper panel FACS plot shows apoptosis of RA SF neutrophils at 0 hour). Apoptosis of neutrophils was measured with Annexin V staining in different culture conditions (media, SF, rIL-9 and anti-IL-9, FACS dot plots of lower panel). (TIF 360 kb) [file 13075_2017_1505_MOESM2_ESM.tif]
